# Immune tolerance and the prevention of autoimmune diseases essentially depend on thymic tissue homeostasis

**DOI:** 10.3389/fimmu.2024.1339714

**Published:** 2024-03-20

**Authors:** Fatemeh Shirafkan, Luca Hensel, Kristin Rattay

**Affiliations:** Institute of Pharmacology, Biochemical Pharmacological Center, University of Marburg, Marburg, Germany

**Keywords:** thymus, autoimmune disease, tolerance, tissue homeostasis, mTEC, thymocytes, Aire, Fezf2

## Abstract

The intricate balance of immune reactions towards invading pathogens and immune tolerance towards self is pivotal in preventing autoimmune diseases, with the thymus playing a central role in establishing and maintaining this equilibrium. The induction of central immune tolerance in the thymus involves the elimination of self-reactive T cells, a mechanism essential for averting autoimmunity. Disruption of the thymic T cell selection mechanisms can lead to the development of autoimmune diseases. In the dynamic microenvironment of the thymus, T cell migration and interactions with thymic stromal cells are critical for the selection processes that ensure self-tolerance. Thymic epithelial cells are particularly significant in this context, presenting self-antigens and inducing the negative selection of autoreactive T cells. Further, the synergistic roles of thymic fibroblasts, B cells, and dendritic cells in antigen presentation, selection and the development of regulatory T cells are pivotal in maintaining immune responses tightly regulated. This review article collates these insights, offering a comprehensive examination of the multifaceted role of thymic tissue homeostasis in the establishment of immune tolerance and its implications in the prevention of autoimmune diseases. Additionally, the developmental pathways of the thymus are explored, highlighting how genetic aberrations can disrupt thymic architecture and function, leading to autoimmune conditions. The impact of infections on immune tolerance is another critical area, with pathogens potentially triggering autoimmunity by altering thymic homeostasis. Overall, this review underscores the integral role of thymic tissue homeostasis in the prevention of autoimmune diseases, discussing insights into potential therapeutic strategies and examining putative avenues for future research on developing thymic-based therapies in treating and preventing autoimmune conditions.

## 1 Introduction

The adaptive immune system relies on the capability to recognize foreign peptides and fight intruding pathogens, thereby providing protection against the infection by viruses, bacteria or fungi. The broad range of foreign peptide recognition depends on the high receptor diversity of the patrolling immune cells. This receptor diversity is key in order to prevent infections, but this mechanism is also increasing the risk of producing immune cell receptors that react towards the host's own peptides and thereby leading to autoimmune diseases. The selection mechanisms that developing T-lymphocytes undergo in the thymus are indispensable checkpoint mechanisms during T cell maturation, leading to the removal of autoreactive T cells by deletion or fate-diversion into regulatory T cells.

Lymphocyte pools are under a steady turnover and get constantly replenished by newly developing cells. The progenitors of T-lymphocytes originate from haematopoietic progenitor cells (HPCs) in the bone marrow (BM) and traffic to the thymus where they differentiate and undergo maturation and selection (i.e. positive and negative selection). This requires the directed migration of the precursor cells via the blood towards the thymus as well as intravascular adhesion and egress from venules into the thymic stroma. Proper thymus function essentially depends on the continuous thymic homing of lymphoid progenitor cells. Even though thymus-resident T cell progenitors have been reported to be capable of maintaining T cell development for several months when the bone marrow is deprived of T cell progenitors (1, 2), the absence of intrathymic competition may lead to T-lineage acute lymphoblastic leukemia (3). The seeding of the thymus during embryonic development (E11.5 in mice and at the 8th week of gestation in humans) is mediated by a vasculature-independent pathway and later in embryonic development and postnatally via a vasculature-dependent pathway (4).

Thymic tolerance induction is mediated by thymic antigen-presenting cells (APCs) including thymic epithelial cells (i.e., cortical thymic epithelial cells (cTECs) and medullary thymic epithelial cells (mTECs)), dendritic cells (DCs) and thymic B cells (5–9). These thymic APCs present endogenously transcribed and imported peripheral peptides by major histocompatibility complex (MHC) class I and II molecules on their surfaces to thymocytes, selecting non-self-reactive thymocytes. Autoimmune diseases develop under circumstances where tolerance towards self-antigens in impaired, either due to a loss of central tolerance selection or impaired peripheral suppressive immune response regulation (10–12). Peripheral tolerance is mediated by CD4^+^CD25^+^FOXP3^+^ Treg (regulatory T cells) which can either develop intrathymically, these Tregs are referred to as natural Tregs (nTregs) or in the periphery after antigen contact which are referred to as induced Tregs (iTregs) (13–15). The pathologies of several autoimmune conditions, such as type 1 diabetes, rheumatoid arthritis, multiple sclerosis, systemic lupus erythematosus and myasthenia gravis (MG) are based on dysfunctional Tregs (16–18). The vast variety of cell types involved in the regulation of thymocyte development and selection, central tolerance induction and Treg development, ranging from B cells, natural killer (NK) cells, Tuft cell, macrophages over endothelial cells and fibroblasts to epithelial cells and dendritic cells keeps increasing as we unveil the mechanisms of central tolerance. In this review we will focus on the importance of thymic tissue homeostasis and the involved cell-cell interactions during thymic immune tolerance induction providing an integrated overview over recent findings and how thymocytes, epithelial cells, dendritic cells, B cells, endothelial cells and fibroblasts interact with each other to maintain self-tolerance.

## 2 Central immune tolerance

### 2.1 T cell migration and cell-cell interaction

The progenitors of T-lymphocytes originate from haematopoietic progenitor cells (HPCs) in the bone marrow and traffic to the thymus where they differentiate and undergo maturation and selection (i.e. positive and negative selection). T cell progenitors are reported to preferentially immigrate at the cortex-medulla-junction (CMJ) of the thymus at venules and then migrate through the thymic cortex and medulla regions and encounter the self-repertoire presented by epithelial and dendritic cells (19–21).

**Figure 1:**
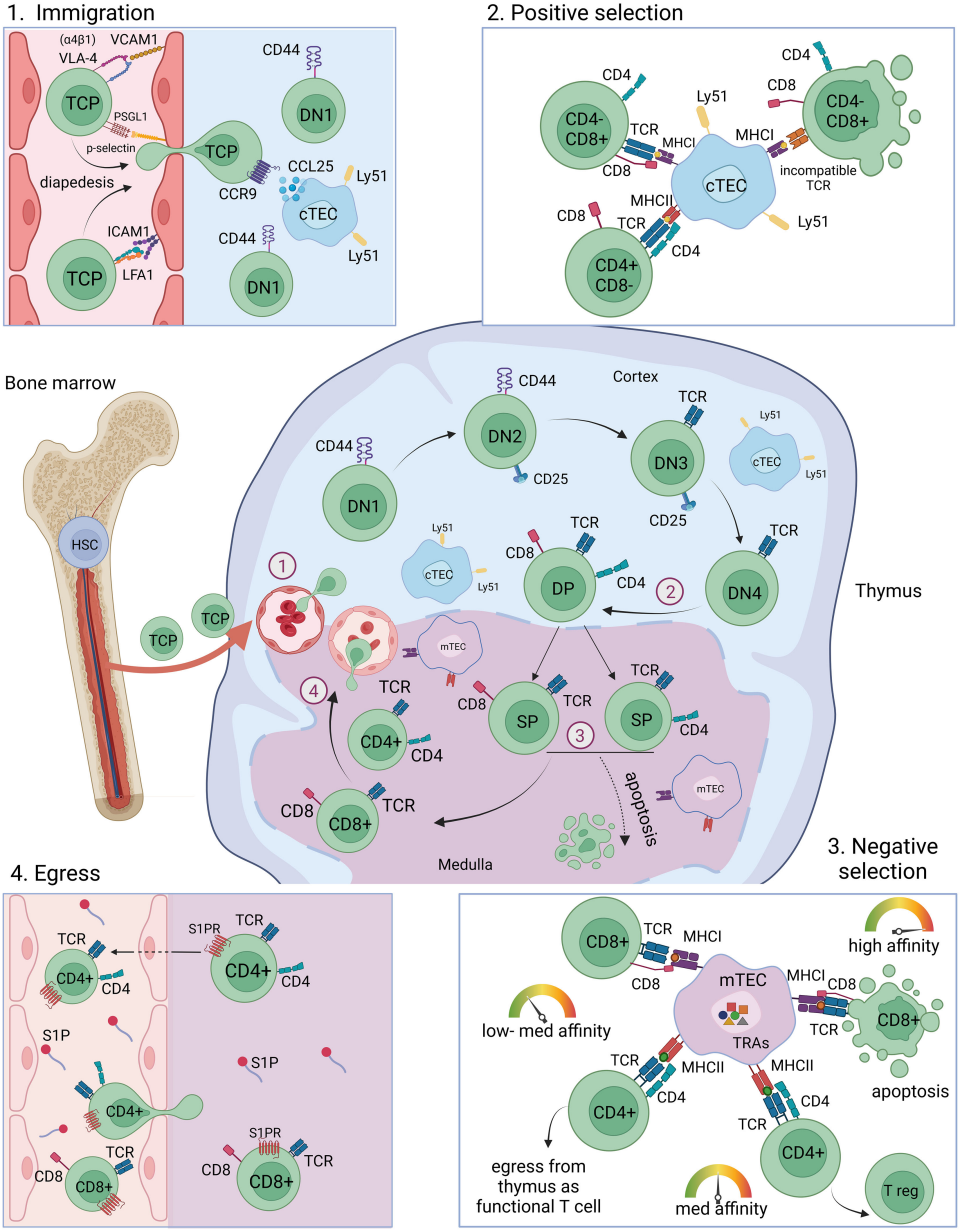
T cell maturation and selection in the thymus. T cell progenitor cells originate from the bone marrow from HSCs and migrate into the thymus through blood vessels at the cortex-medulla-junction (immigration). Depicted is one lobule of the thymus. The maturation from DN1-DN2-DN3-DN4 into DP thymocytes takes place preferentially in the cortex region of the thymus, where cTECs mediate positive selection of DP thymocytes. SP thymocytes migrate into the medulla where they encounter self-peptides presented by mTECs and DCs and undergo negative selection. High reactivity towards self-antigens leads to apoptosis, intermediate reactivity towards self-antigens leads to the development into Tregs and low reactivity towards self-antigens leads to the maturation of CD4^+^ and CD8^+^ mature naïve T cells which egress from the thymus through blood vessels. T cell migration towards the blood vessels and egress is facilitated by S1P gradients. HSC, haematopoietic stem cell; TCP, T cell progenitor; DN, double negative; DP, double positive; SP, single positive; cTEC, cortical thymic epithelial cell; mTEC, medullary thymic epithelial cell; TRA, tissue restricted antigen; MHC, major histocompatibility complex; TCR, T cell receptor; Tregs, regulatory T cells; S1P, spingosine-1-phosphate.

Intercellular communication among APCs, endothelial cells (ECs) and developing T cells is key to proper thymic tissue homeostasis and central immune tolerance induction. The lympho-stromal crosstalk between stromal cells and thymocytes is known to be essential for T cell development and thymic epithelial cell maintenance (22, 23) (**Figure 1**). The bilateral regulation of migrating thymocytes and stromal cells is an intertwined dependency of high clinical relevance in which the immune-metabolic interactions in the thymus are changing in an age-dependent manner (24). A better understanding of the involved interactions and signaling pathways is a prerequisite to be able to develop potential future treatments. Of particular interest are the involved recruiting and guiding signals for thymocyte migration. Homing of T cell progenitors to the thymus has been demonstrated to rely on a multistep adhesion cascade involving selectin ligands, chemokine receptors and integrins, which is similar to, but molecularly distinct from the adhesion cascades that guide leukocytes to lymph nodes or the bone marrow (25). Among the chemokines, CCL21 and CCL25 and their respective receptors, CCR7 and CCR9, were shown to be involved in fetal thymus colonization (26–30). During adult thymus seeding several lymphocyte-endothelial cell interactions such as P-selectin glycoprotein ligand (PSGL-1)/P-selectin, α4β1/VCAM-1, LFA1/ICAM-1 as well as lymphocyte-epithelial cell CCR9/CCL25 interactions were shown to be involved (25, 31). For instance, the CCR9 ligand CCL25 (TECK), which plays a key role in the multi-step adhesion cascades that guides leukocytes to the thymus, is expressed by medullary and cortical epithelial cells (32, 33). Thymic immigration and egress of T cell progenitors and mature T cells was proposed to occur mainly in post-capillary venules at the cortex-medulla-junction based on electron microscopy studies and an ex vivo study using CFSE-labeling of cells (19). These *ex vivo* studies could demonstrate the localization of T cells in close proximity to venules, however, it was not possible to determine whether the cells were entering or exiting the thymus. *In vivo* intravascular staining of CD4 SP T cells showed a preference for a transmigration at the CMJ, labeling presumably egressing cells (34).

Thymocyte migration in the thymus is dependent on sphingosine-1-phosphate (S1P) signaling which is essential for the egress of mature thymocytes (34–36). The substrate for S1P synthesis is sphingosine, which is phosphorylated by sphingosine kinase 1 (Sphk1) or sphingosine kinase 2 (Sphk2) to generate S1P. Degradation on the opposite is mediated by S1P phosphatase (SPP), S1P lyase 1 (Sgpl1), S1P lyase 2 (Sgpl2) or phospholipid phosphatase 1, 2, or 3 (Plpp1, Plpp2, Plpp3). After negative selection in the thymus medulla, mature T cells upregulate the sphingosine-1-phosphate receptor 1 (S1PR1) making them responsive to S1P gradients within the tissue guiding them to egress from the thymic tissue. Premature S1PR1 expression in thymocytes is associated with the development of autoimmunity, probably due to incomplete negative selection and the egress of self-reactive T cells (34). Red blood cells are a major source of S1P in the blood (37). Another source of S1P within the thymic tissue are pericytes (34). Further, endothelial cells are implicated in the regulation of S1P gradients, as lymphatic endothelial expression of the S1P transporter Spns2 was shown to be crucial for peripheral T cell migration and affects thymic T cell egress (38–41). Local S1P secretion by endothelial cells is thought to be a crucial guiding cue for T cell egress from the thymus (42).

Beyond playing a role in thymocyte immigration and egress, EC-derived factors were shown to be involved in the developmental progression during thymocyte maturation. The EC-derived factor Kit ligand (KitL, also called stem cell factor (SCF)) is essential for early thymocyte proliferation (43). Membrane-bound KitL (mKitL) is expressed by cortical thymic epithelial cells and endothelial cells in the cortex (44). Interestingly, thymocyte development at the DN1 stage was shown to depend on EC-derived mKitL, whereas the consecutive stage of DN2 was shown to depend on cTEC-derived mKitL. Additionally, EC-derived factors were demonstrated to play a crucial role in thymic tissue regeneration in adult mice. The ability of the thymus to undergo tissue regeneration after damage caused by infection, chemotherapy or radiation is critical in order to prevent T cell deficiency associated health conditions. Following radiation-induced damage, thymic endothelial cells secrete bone morphogenetic protein 4 (BMP4) which leads to the upregulation of *Foxn1* expression in thymic epithelial cells and increased expression of Foxn1 target genes such as delta-like ligand 4 (DLL4) (45). Foxn1 target genes are involved in the regulation of TEC development, maintenance and regeneration. Moreover, multi-organ dataset analysis shown that neighboring cells can induce tissue-specific behavior in endothelial cells, the other way around endothelial cells can affect the gene expression programs of adjacent cells. Examples for such cross-influence are described for cardiac endothelial cells that were reported to express cardiac muscle-specific genes. Another example are sinusoidal endothelial cells that were shown to express genes usually expressed by hepatocytes (46, 47).

### 2.2 Thymic epithelial cells and self-tolerance

**Figure 2:**
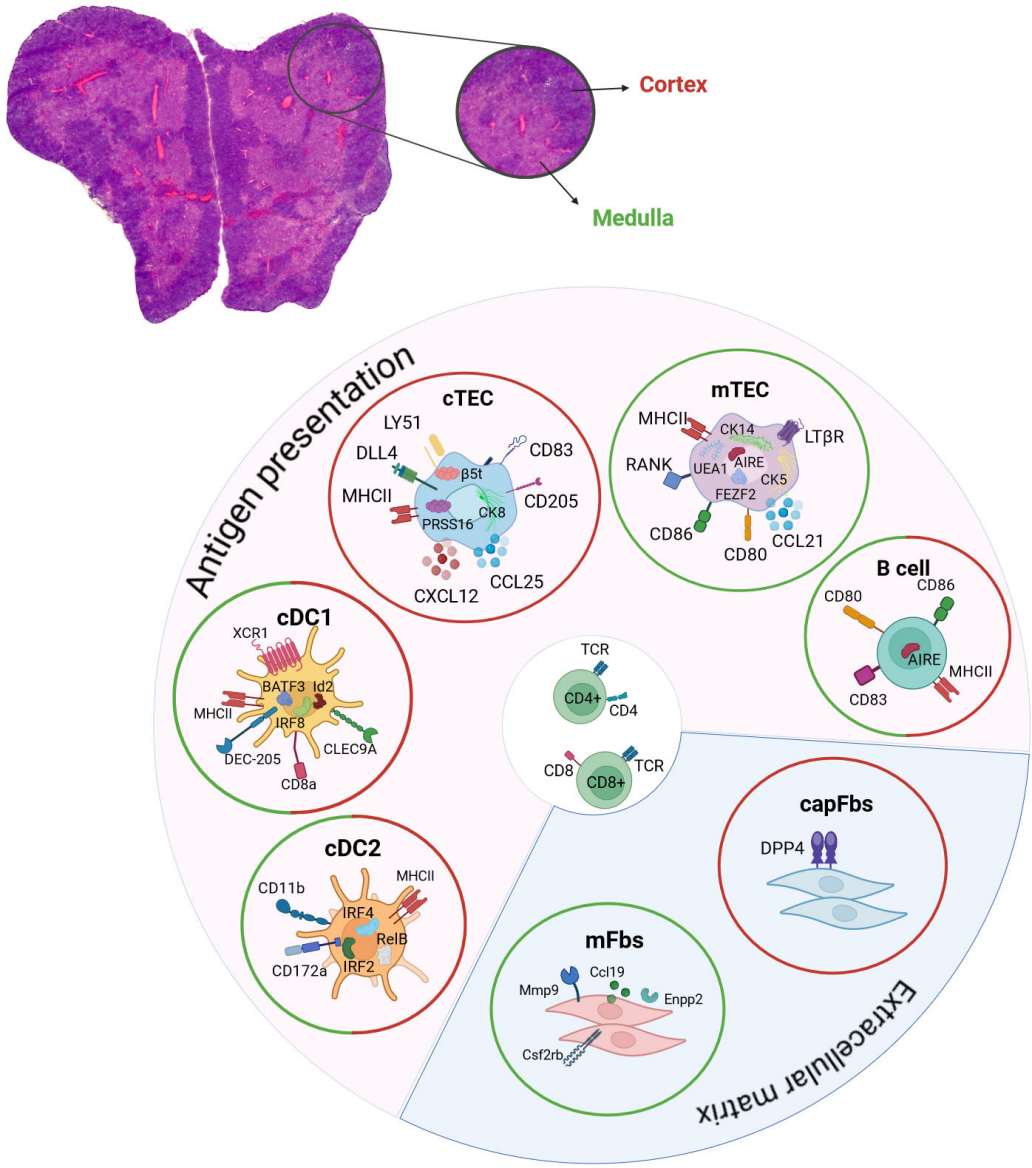
Contribution of DCs, TECs, B cells and fibroblasts to central tolerance induction. Hematoxylin and eosin staining of a mouse thymus. Depicted are the two lobes of the thymus and the two compartments of outer cortex (red) and inner medulla regions (green). The respective expression of chemokines, surface and intracellular markers that are characteristic for cDC1, cDC2, mTECs, cTECs, thymic B cells, capFbs and mFbs are shown. The contribution to antigen presentation and the extracellular matrix in the thymus is indicated. The location of cDC1, cDC2, mTECs, cTECs, thymic B cells, capFbs and mFbs in the cortex (red circle) and/or medulla (green circle) are shown. cDC, conventional dendritic cell; cTEC, cortical thymic epithelial cell; mTEC, medullary thymic epithelial cell; capFbs, capsular fibroblasts; mFbs, medullary fibroblasts.

The thymus consists of two lobes which are structured into two different compartments, the outer cortex region and the inner medulla region (**Figure 2**). Thymic epithelial cells are morphologically distinct to epithelial cells of other tissues in the body, where cuboidal, squamous or columnar morphologies that are organized in polarized mono- or multilayers are the predominant features (48). In contrast, thymic epithelial cells build a 3D meshwork of epithelial cells permeated by endothelial cells and populated by T cells, dendritic cells and thymic B cells (49–52). Cortical thymic epithelial cells show a characteristic structure of flattened ovoid cells forming long looping structures, engulfing developing thymocytes. Whereas cTECs are described to show a radially oriented alignment towards the capsule, mTECs are positioned in the medulla seemingly without a clear orientation or polarization. Moreover, the mTEC morphologies are highly diverse and no definitive pattern of the mTEC structure could be detected in an elegant study using Confetti (Brainbow2.1) mice labeling of individual cells and analysis by confocal microscopy (53).

The endoderm-derived epithelial cells in the thymus are differentiated into functionally distinct cortical thymic epithelial cells (cTECs) in the cortex and medullary thymic epithelial cells (mTECs) in the medulla, each having specific function during the sequential T cell developmental steps. The different stages of T cell development are well studied (54, 55) and are reviewed elsewhere in detail (56–60). In brief, double-negative (CD4^-^CD8^-^) thymocytes migrate through the cortex and undergo T cell receptor gene recombination. The newly generated TCRs are subsequently subjected to a selection process, called positive selection, in which the newly generated pool of thymocytes is tested for TCRs that are capable to bind and recognize MHC on antigen-presenting cells such as cTECs, providing a survival signal to the developing thymocyte (8, 61, 62). Positively selected thymocytes further develop into single-positive CD4^+^- and CD8^+^-T cells which are undergoing a second selection step, called negative selection, in which T cells carrying TCRs with high reactivity towards self-antigens are depleted from the T cell population. Intermediate binding intensities at this selection step will lead to the development into Foxp3-expressing CD4^+^ regulatory T cells (Tregs) (**Figure 1**) (63). The presentation of self-peptides and mediation of negative selection is accomplished mainly by mTECs as well as by DCs and thymic B cells in the medulla region of the thymus (5–9). The endogenous self-antigen expression by mTECs is referred to as promiscuous gene expression (pGE) or ectopic gene expression.

One of the main characteristics of promiscuous gene expression is the so-called mosaic expression pattern of tissue restricted antigens (TRAs). Each TRA is expressed in about 1-5% of the mTECs at a certain point in time. This characteristic is conserved between mouse, rat and human (64–69). Despite the high degree of variance at the individual cell level, the mTEC population as a whole manages to reflect the entire self-repertoire (70–74). Due to the high heterogeneity of thymic self-peptide expression the cellular and molecular regulations involved remain challenging to identify (75–80). The promiscuously expressed genes in mTECs comprise a diverse range of biological functions and tissue specificities and vary greatly in their regulatory elements and promoter regions. Notably, in the context of thymic self-antigen expression by mTECs, several peripheral tissue-specific transcription factors were shown to be dispensable for the respective thymic gene expression (69, 81, 82). In the past, several studies focused on investigating the regulatory mechanisms of the self-antigen mosaic expression patterns evaluating to which degree this phenomenon arises based on stochastic or regulated processes. Originally, studies performed on bulk mTEC populations were unable to detect recurrent gene expression patterns (66, 69). Likely, due to the high heterogeneity in gene expression from cell-to-cell, the patterns of subpopulations were not detectable on the population level at the time. However, the development of single cell sequencing technology and methods for selective enrichment of TRA-expressing mTEC subsets enabled the detection of recurring gene expression patterns in mTECs in mouse and human, thus arguing for yet unidentified regulatory mechanisms rather than a solely random process (67, 68, 70, 71, 73). Early during embryonal development, the thymus is developing through clonal expansion (83). Different developmental models of how the co-expression patterns of promiscuous gene expression evolve postnatally, such as a lineage diversification model and a linear developmental program model have been proposed (67, 68). Distinct conserved groups of self- antigens that are co-expressed in mTECs might share regulatory features that establish sub-lineages of mTECs. Alternatively, the co-expressed groups of self-antigens might be the result of a developmental program over the lifespan of a mTEC. This would lead to different sets of self-antigens being expressed and presented over time and an agile representation of the self-repertoire in the thymus. Interestingly, based on ex-vivo culturing experiments the linear developmental model is more likely to be accurate for the mature mTEC compartment (67). In difference to the early and mature mTEC populations, late developmental stages are more likely to develop based on lineage diversification into sub-lineages. The late mTEC stages comprise of post-Aire cornified (Krt10^+^) mTECs (84, 85) as well as Tuft-mTECs (Dclk1^+^) (72, 77, 86, 87), microfold mTECs (Gp2^+^), enterocyte/hepatocyte mTECs (Hnf4^+^), keratinocyte-like mTECs (Grhl^+^), neuroendocrine mTECs (Foxa+), cilitated mTECs (Foxj1^+^) and other mimetic mTECs (88, 89). Previous reports on thymic cell populations that represent characteristics of peripheral differentiated cell types and expression of mimetic cell genes in the thymus were initially difficult to distinguish from features of promiscuous gene expression of peripheral antigens, however single-cell technologies and high throughput assays enabled a revised view on those findings, leading to the identification of several distinct mimetic cell populations in the thymus with characteristic gene expression profiles, lineage-specific transcription factors, phenotypical characteristics and probably specific functions in the thymus reviewed in other review articles in more detail (90, 91). The mature mTEC population is subject to high turnover with a half-life of 12-14 days (92, 93), thus leading to a constant change in self-antigen co-expression groups over the course of mTEC development and lifetime. The mTEC population is not synchronized in their development, thus at any given age of the thymus different developmental stages of mTECs can be found. Thereby the two variables of I.) different self-antigens being expressed at different developmental timepoints and II.) the spatial differences within the thymic tissue at which developmental step the mTECs currently are leads to a highly versatile and heterogeneous presentation of the self-repertoire to developing T cells. The migration speed and distance of thymocytes as well as the number of contacts with APCs are defining factors in the efficiency of self-tolerance induction. Several studies assessed the migration and selection characteristics of developing T cells in the thymus using ex vivo culturing of tissue sections, reaggregation of thymic tissue or thymic explants (94, 95). *In vivo* microscopy of murine thymi is difficult first due to the opaqueness of the tissue which limits the penetration and visibility in two-photon microscopy and second due to its positioning on top of the moving heart, which makes *in vivo* analysis difficult. The interaction or contact time during positive selection between thymocytes and stromal cells was analyzed in a three-dimensional thymic organ culture and identified to be of either “dynamic” short interactions of 13-36 minutes or “stable” longer interactions of 6-12 hours (96). The migration speed of thymocyte was analyzed using ex vivo two-photon microscopy analysis of thick thymus sections before and after antigen encounter (97). The interval speed before antigen contact was about 11 µm/min and 9 µm/min after antigen contact. SP thymocytes are estimated to reside in the medulla region of the thymus for about 4-5 days as measured using BrdU-labelling, intrathymic injection of a cellular tag or RAG2p-GFP reporter mice (98). Elimination of self-reactive T cells upon self-antigen encounter and strong affinity towards self is executed swiftly, resulting in a rapid calcium influx and migratory arrest within a few minutes followed by caspase 3 activation and thymocyte death after a few hours (97). Imaging of ex vivo agarose-embedded sections of thymic explants was used in another study of transgenic reporter mice labelling thymocytes and DC interactions during negative selection in the medulla region (99). Accelerated migration rates of 10-20 µm/min were detected for thymocytes migrating towards the medulla after positive selection and within the medulla during negative selection in this setting. Further, short dynamic contacts between DCs and thymocytes of 1-2 minutes with multiple contacts per hour were described in this setup. The migration range that thymocytes travel was shown to be limited to 30-50 µm in total which was surprising due to the previously expected scanning of T cells with multiple APC contacts before egressing the thymus (100). It is important to note, that different experimental setups with different temperatures will result in differences in the observed migration speed and distance of cells and hence has to be kept in mind when analyzing ex vivo imaging analysis or imaging setups where the thymus is exposed from the chest of mice.

The transcriptional and epigenetic regulation of self-antigen expression in mTECs might in difference to the respective gene regulation in peripheral tissues be dependent on different thymus-specific regulatory mechanisms (69, 81, 82). Two factors regulating mTEC development and affecting the expression of self-peptides in mTECs are the transcriptional regulator Autoimmune regulator (Aire) and the transcription factor Forebrain Embryonic Zinc Finger-like protein 2 (Fezf2) (76, 78). Aire is a transcriptional regulator known to be essential for mTEC maturation and T cell selection (85, 101–103). Aire-deficiency leads to impaired thymocyte selection due to missing tissue restricted antigen (TRA) expression by mTECs and results in the development of autoimmune symptoms in Aire-deficient mice and patients with mutations in the AIRE gene (76, 104, 105). The loss of AIRE is one of the prominent examples for monogenic autoimmune disorders in which the loss of central tolerance is causative for the development of the autoimmune disorder autoimmune polyendocrinopathy candidiasis ectodermal dystrophy (APECED) (or Autoimmune polyendocrine syndrome type 1 (APS1)) leading to multiorgan autoimmunity (106). The mode of action of the transcriptional regulator Aire is at the epigenetic level, by acting on transcriptional elongation and by acting on superenhancers (107, 108). Aire is interacting with a multitude of different factors identified so far, including proteins involved in nuclear transport (HDAC1, HDAC2) (109), chromatin-modifiers (H3K4me0) (110, 111), transcriptional regulators (RNA PolII, DNA-PK, P-TEFb, HNRNPL, BRD4) (102, 112–116), and proteins involved in mRNA processing (JMJD6) (117) which is reviewed in other articles in more detail (91, 101, 118). The mode of action of the transcription factor Fezf2 on the other hand, is through consensus binding site recognition. The consensus sequence for the Fezf2 TFBM in mice was recently identified based on Fezf2 ChIP-seq experiments and a Stamp-based comparison to the Fezf2 TFBM in zebrafish (119). Fezf2-deficiency in mice was shown to lead to disturbed TRA expression and negative selection leading to the development of autoimmune symptoms (78). Recently, Fezf2 was identified to regulate late mTEC development and thymic Tuft cell development (119). Aire and Fezf2 are regulating distinct sets of self-antigens in mTECs and might likely reflect regulatory mechanisms at different timepoints during mature mTEC development or of sublineages of mature mTECs. Noteworthy, these two regulatory factors act relatively broadly on mTEC development and maturation and regulate the gene expression of several thousands of genes. Possibly, Aire and Fezf2 might be involved in the regulation of a downstream regulatory network of transcription factors that are regulating self-antigens leading to the particular expression patterns of TRAs in a mosaic expression pattern (119). Further factors involved in the regulation of those recurring gene expression patterns in mTECs remain to be identified. The question arises as to which degree Aire and Fezf2 are specific transcriptional regulators of self-antigen gene expression or whether their impact on mTEC development and maturation indirectly affects self-antigen expression. To this end, a recent study analyzed promiscuous gene expression and Aire-dependent gene expression over the course of aging using single-cell RNA-sequencing analysis and lineage-tracing showing, that AIRE-controlled genes were downregulated during mTEC development despite the continued expression of *Aire* and *Fezf2*, hence different mechanisms of transcription reliant on other regulators might be involved (120). The regulation of Aire itself and the mechanisms through which it regulates gene expression in mTECs has been intensively studied. One of the interaction partners and regulators of Aire is Hipk2, which is involved in regulating the activity of Aire. Hipk2-dependent phosphorylation was shown to inhibit the co-transcriptional activator function of Aire (121). Further, the protein deacetylase Sirtuin-1 (Sirt1) was shown to be involved in Aire-dependent gene regulation and induction of immunological self-tolerance by deacetylation of Aire (122). Additionally, the acetyltransferase KAT7 was identified to be important for Aire-dependent gene expression and tolerance induction by increasing chromatin accessibility at Aire-induced gene loci (123). Other posttranslational modifiers of Aire such as CBP, FBXO3, PIAS1 (124–126) and factors which are involved in the regulation of the Aire gene such as IRF4, IRF8, TBX21, TCF7 and CTCF were identified (127).

Besides the regulatory role of Aire in the promotion of mTEC maturation and promiscuous gene expression, Aire was also shown to be involved in chemokine production, the positioning of DCs within the thymus and extrathymic Aire functions early during embryogenesis are described (128). Aire deficiency leads to the reduced expression of the CCR4 and CCR7 ligands which are expressed by mTECs and are involved in thymocyte migration within the tissue, being CCL5, CCL17, and CCL22 for CCR4 and CCL19 and CCL21 for CCR7 (129). The mTEC-DC interaction of XCR1 on DCs and XCL1 on mTECs is important for the positioning of thymic DCs in the medulla and Aire-deficiency leads to a lack of XCL1 expression in mTECs, a lack of DCs in the medulla region, defective generation of Tregs and the development of autoimmune dacryoadenitis (130). Aire has also been described to be expressed in other tissues such as lymph node and spleen, in cells, termed extrathymic Aire-expressing cells (eTACs), which are of hematopoietic origin, exhibit DC-like features and express Aire-regulated genes that are distinct to thymic TRAs (131–133).

Besides Aire and Fezf2 other transcription factors are important for the regulation of mTEC gene expression and mTEC development. Insulinoma-associated 1 (Insm1), a zinc finger protein was shown to be expressed in Aire-expressing mTECs and neuroendocrine mimetic cells (FoxA+ mimetic cells) and is involved in the regulation of Aire expression, TRA expression and tolerance induction (134). The zinc finger transcription factor Ikaros (*Ikzf1*) regulates Aire^+^mTEC development, thymic mimetic cell development, TRA expression and immune tolerance induction (135). The absence of Ikzf1 led to an expansion of Dclk1^+^ tuft cells and a reduction in Gp2^+^ microfold cells, hence altering the composition of the mimetic cell populations in the thymus. Additionally, the transcription factors Ehf, Klf4 and Elf3 were shown to be involved in the regulation of gene expression in mTECs (119). The complexity of the medullary thymic epithelial cell population and the heterogeneity in gene expression in mTECs have historically made it challenging to identify the regulatory mechanisms. Notably, advancements such as single-cell sequencing have enabled us to gain an increasingly better understanding of these regulatory mechanisms.

Beyond the transcriptional regulation of promiscuous (or ectopic) gene expression other factors are influencing the efficiency of central tolerance induction. Among these, the MHCII-TCR interaction between thymic APCs and thymocytes, referred to as thymic crosstalk, influences mature mTEC development, TRA expression and immune tolerance induction, leading to autoimmune phenotypes if abrogated (22, 23, 136). Moreover, the butyrophilin surface receptor Btn2a2 expressed on thymic epithelial cells is important for central T cell tolerance induction (137, 138). Btn2a2-deficient mice showed enhanced effector CD4^+^ and CD8^+^ T cell responses, impaired CD4^+^ regulatory T cell induction, potentiated antitumor responses, and exacerbated experimental autoimmune encephalomyelitis (137). While another study reported increased TCR signaling and CD5 levels for developing thymocytes in Btn2a2-deficient mice with otherwise unchanged TEC compartments and thymic T cell populations (138).

Notably, defects in the expression of specific self-antigens by mTECs can predispose to the onset of organ-specific autoimmune diseases. One example is the development of autoimmune diabetes in cases where the thymic insulin (Ins2) expression is abrogated (139–141). The development of type 1 diabetes is associated with a causal polymorphism in the insulin gene that leads to reduced thymic expression and the occurrence of autoreactivity (141). Other examples are the development of uveitis when the expression of interphotoreceptor retinoid-binding protein (IRBP) in thymic epithelial cells was faulty (142) and the development of autoimmune myocarditis in mice and human upon loss of α-isoform of myosin heavy chain (α-MyHC) gene expression by mTECs (143). Moreover, variants in the gene encoding the α-subunit of the muscle acetylcholine receptor (Chrna1), which is the main target of pathogenic auto-antibodies in autoimmune myasthenia gravis, caused reduced thymic expression and is associated with the development of the disease (144).

### 2.3 Thymic fibroblasts, B cells and dendritic cells

Mesenchyme-derived thymic fibroblasts can be differentiated into medullary (mFbs) and capsular (capFbs) fibroblasts based on their location in the thymus and the expression of Dpp4, which is expressed by capFbs but not by mFbs (145, 146). Due to the expression of the matrix metalloprotease (Mmp9), different regulators involved in cell migration (Ccl19, Enpp2, Cd300lg) and cytokine receptors (Csf2rb, Csf2rb2) by mFbs a role in the regulation of cell migration in the medulla was postulated. Interestingly, the development of mFbs is dependent on SP thymocyte population of the thymus, but independent of mTEC development. Conditional knock-out of LtßR in fibroblasts led to autoimmune phenotypes in lung, pancreas, salivary gland and liver, identifying thymic fibroblasts as one of the receptor-bearing target cells for thymic lymphotoxin-dependent signaling and their contribution to central tolerance induction (145). Fibroblasts play an important role in the production of the extracellular matrix (ECM) in diverse tissues and are implicated in tissue repair and healing processes (147). Tissue damage induced secretion of IL-25 by thymic tuft cells and IL-33 by fibroblasts leads to the activation of ILC2 (innate lymphoid cell type 2) production of amphiregulin and Il-13 and tissue regeneration in a dexamethasone-induced acute thymic involution mouse model (148).

Thymic B cells are one of the antigen-presenting cells in the thymus that are involved in the mediation of negative selection (5). Two types of thymic B cells are described, resident B cells, which develop intrathymically from early fetal liver derived progenitor cells (149–152), and circulatory B cells which immigrate from the periphery and acquire a specific thymus-resident B cell phenotype, including the expression of *Aire* (5, 153). Different localizations of thymic B cells have been described with B cell progenitors being preferentially localized in the thymic cortex, whereas mature B cells are primarily found in the corticomedullar and medullary regions (151) and B cells that are found in perivascular spaces (154, 155). The age-associated increase in the frequency of thymic B cells was attributed to an increase in peripheral-derived B cells in the thymus with a preferred localization in perivascular spaces. Further, B cells which are localized to the perivascular spaces express CD21, CD72, CD37 and the chemokine receptor CXCR3 (154, 155). Despite the increase in thymic B cell numbers upon aging, thymic B cells were found to exhibit lower expression of MHCII, CD80, CD83, CD86, Aire and Aire-dependent genes in adult and aged mice compared to young mice (154, 156). These age-dependent changes during the course of aging offer the possibility, that also the reduction of thymic B cells as one type of thymic APCs, similarly to the age-dependent alterations in mTEC physiology, can contribute to the phenomena of thymic involution and impaired thymopoiesis in aged thymi. Thymic hyperplasia is often observed in patients with myasthenia gravis. The thymic architecture in patients with myasthenia gravis and thymic hyperplasia is disturbed and B cell follicles containing active germinal centers (GCs) are formed, which give rise to plasma cells and are a source of autoantibodies. Increased chemotactic signal through CXCL13 and a higher release of the B cell activating factor (BAFF) by thymic epithelial cells are thought to promote B cell recruitment to the thymus in myasthenia gravis (153, 157, 158). In difference to myasthenia gravis associated hyperplasia of the thymus, patients with systemic lupus erythematosus show thymus atrophy with disorganized medulla regions, diminished cortex regions and enlarged perivascular spaces with lymphocytic follicles and plasma cells (153, 159).

The significance of dendritic cells in initiating immunological tolerance was demonstrated through mutant mouse models. In these models, the lack of DCs led to compromised negative selection of thymocytes (160, 161). Thymic dendritic cells consist of subsets that are distinct both phenotypically and functionally, being conventional DCs (cDC1 and cDC2), plasmacytoid DCs (pDCs), and monocyte-derived DCs (moDCs) or activated DCs (sDCs) that are either derived from intrathymic progenitors or are migrating towards the thymus from peripheral tissues (162). Murine conventional DCs are distinguished into cDC1 and cDC2 populations based on surface marker expression and their distinct developmental regulators. Thus, cDC1s are identified by their surface expression of XCR1, CD8α, CLEC9A, and DEC205, and they are developmentally dependent on IRF8, Id2, and BATF3 (163). CD8α ^+^CD11b^–^ cDC1s comprises the majority of cDCs in the murine thymus. Most cells in this DC subset are thought to originate intrathymically from a common lymphoid progenitor (CLP) (164–166). Similar to CD8α^+^ cDCs in the periphery, thymic CD8α^+^CD11b^–^ cDC1s are very efficient at cross-presenting mTEC-derived antigens on MHC class I, which was shown using *in vitro* experiments (167–169). *In vivo*, thymic cross-presentation of self-antigens by DCs contributes to the induction of CD8^+^ T cell tolerance, however, the identity of the cross-presenting DC subset or the contribution of different subsets is currently not sufficiently addressed and necessitates further analysis (169, 170). Instead, cDC2s are defined by their surface expression of CD11b and CD172a (SIRPα), are developmentally dependent on IRF4, IRF2 and RelB, and preferentially activate CD4^+^ helper T cell responses (171, 172). Thymic cDC2s and pDCs are migrating towards the thymus from the periphery, importing peripheral antigens for central tolerance induction (173, 174). Moreover, thymic epithelial cells and thymic dendritic cells contribute non-redundant antigens to facilitate the process of negative selection (175) by using different antigen processing machineries, leading to the generation of distinct self-peptides. The self-antigen diversity presented by mTECs is further enhanced due to mechanisms of mis-initiation, alternative splicing and the expression of endogenous retroelements which increase the self-peptidome presented to developing T-lymphocytes (176). Thereby DCs and mTECs represent the self-repertoire presented in the context of thymic negative selection in an integrated manner.

Additionally, a specific group of dendritic cells in both murine and human thymi, termed transendothelial dendritic cells (TE-DCs), has been identified for their unique location within the vascular walls of thymic microvessels (9). From these positions, TE-DCs extend their cellular processes into the bloodstream to gather circulating antigenic materials. The unique transendothelial placement of these antigen-sampling TE-DCs has been found to rely on the interaction between the chemokine receptor CX3CR1, expressed on the DCs, and its ligand CX3CL1, which is abundantly present in thymic capillaries. Furthermore, TE-DCs are capable of capturing and cross-presenting blood-derived proteins, leading to the specific deletion of developing antigen-specific T cells. Together, the different thymic APC populations, achieve to present the self-repertoire in an astonishingly comprehensive manner, ensuring the successful selection of newly generated T cells to continuously generate non-self-reactive naïve mature T cells to replenish the peripheral T cell pool.

## 3 Thymus development, genetic defects and autoimmune diseases

During the, 1960s, the functional role of the thymus was revealed. Until then the thymus itself and the presence of lymphocytes in the thymus was described, however, the thymus was thought of as a terminal storage or graveyard for lymphocytes and its actual role was unknown at that point (177). The immunological role and the importance of the thymus for survival and its tolerogenic role were described by J. F. A. P. Miller (178, 179). The removal of the thymus (i.e. thymectomy) in mice at the age of 3 weeks was shown to lead to the development of autoimmunity (180). Positive selection was first described by M. J. Bevan (181) and the process of intrathymic clonal elimination for tolerance induction was described 10 years later (182, 183). The importance of the thymic epithelium in tolerance induction was identified using mouse chimeras (184) and finally, the identification of promiscuous gene expression of self-peptides in mTECs was described in, 2001 by J. Derbinski, A. Schulte, B. Kyewski and L. Klein (185) after several individual reports on genes that were found to be expressed by the thymic epithelium (141, 186–189). Since then, research on the thymus and the immunological processes taking place in the thymus has increased immensely. Several factors were identified to be essential for thymus development and the prevention of autoimmune reactions, most importantly the transcription factor Foxn1 (forkhead box protein 1) which is essential for thymic epithelial cell development and consecutive T cell progenitor recruitment (190). Nude mice with a mutation in the Foxn1 gene have congenital athymia and show severe immunodeficiency besides their name giving deficiency in hair growth (191). Athymic nude mice are used as a model system to study thymus development and the development of autoimmune reactions in thymic transplantation experiments. The Foxn1 gene promoter is also widely used in conditional knock-out systems to delete and study gene functions in thymic epithelial cells by crossing Foxn1-cre lines to the “gene of interest”-flox mouse line. Other transcription factors and signaling pathways involved in thymus morphogenesis are reviewed elsewhere (192).

Several monogenic autoimmune diseases have been described to date, in which proper thymic development of the thymic stromal compartment or thymocytes is abrogated. The autoimmune polyendocrinopathy syndrome type 1 (APS1), also known as autoimmune polyendocrinopathy candidiasis ectodermal dystrophy (APECED), is a monogenetic autoimmune disease affecting multiple organs. It is characterized by a mutation in the autoimmune regulator (AIRE) gene, leading to a disruption in central immune tolerance (193). Similarly, autoimmune lymphoproliferative syndrome (ALS) is another monogenic autoimmune condition, distinguished by an accumulation of polyclonal double-negative (DN) T cells. This accumulation results from mutations in either Fas or Fas ligand, or in caspases that are part of the Fas signaling pathway (194). Additionally, the monogenic autoimmune disorder IPEX (immunodysregulation polyendocrinopathy enteropathy X-linked syndrome) arises due to a defect in the Foxp3 gene, leading to a loss of Tregs (CD4^+^CD25^+^) and consequently, a breakdown in peripheral tolerance (195). The identification and characterization of such monogenic autoimmune diseases in mouse and human have been crucial to identify the mechanisms involved in central tolerance induction.

In the course of aging the thymus tissue is undergoing gradual atrophy, a process called involution. Thymic involution occurs in mice and humans and starts approximately at young adulthood or during puberty at the age of about 8-10 weeks in mice and 15-20 years in humans (93, 196). In the context of thymic involution different models are discussed as to whether the process is initiated by a reduction of thymocyte-derived signals or reduced TEC-derived signaling (120, 197–200). Initially a reduced production of progenitor cells originating from the haematopoietic stem cells (HSCs) in the bone marrow and reduced numbers of early T cell progenitors (ETPs) entering the thymus were proposed as initial changes at the onset of involution (201). However, recruitment of progenitor cells into the thymus does not appear to be the sole initiating factor for age-dependent thymic involution, as the ability of aged thymi to recruit intravenously injected lymphoid progenitor cells was shown to be similar to that of young thymi (200). Furthermore, fetal thymic transplants under the kidney capsule of aged mice led to reestablished normal thymic lymphopoiesis and thymocyte subpopulations, including ETPs, DN subsets, DP, and CD4^+^ and CD8^+^ SP T cells. Thus, demonstrating that haematopoietic progenitor cells of aged mice were sufficiently capable to home and proliferate in the young thymic tissue. Conversely, intrathymic injection of ETPs isolated from young mice into old mice did not restore normal thymic lymphopoiesis (200). Instead of a reduction in progenitor cells, changes in the thymic microenvironment and tissue homeostasis came into focus as causative factors for the age-dependent thymic involution. Aged thymi show reduced numbers of cTECs and mTECs and an increase in adipocytes and perivascular spaces (196, 199, 200). Further, a shift in the composition of the TEC population from primarily perinatal cTECs (1-week-old mice) over mature mTECs (4-week-old mice) to mature cTECs and intertypical TECs (showing mTEC and cTEC expression features) (from 16 weeks onwards) can be observed in mice of different ages (120). Furthermore, changes in the gene expression profiles of aged mTECs can be observed, with genes involved in inflammatory signaling, apoptosis and KRAS signaling being upregulated, whereas genes involved in cholesterol homeostasis and oxidative phosphorylation get downregulated (120). The expression of the transcription factor Foxn1, which is essential for TEC development, is reduced in aged thymi, alongside with a downregulation of Foxn1-target genes (202, 203).

Crucially, in aged mice with fully involuted thymi, the upregulation of FOXN1 in thymic epithelial cells (TECs) triggered thymic regeneration. This regeneration was marked by the activation of genes essential for TEC development or for T cell differentiation and repertoire selection. Key genes involved include Delta-like 4 (Dll4), chemokine (C-C motif) ligand 25 (Ccl25), kit ligand (Kitl), and chemokine (C-X-C motif) ligand 12 (Cxcl12). This activation led to enhanced thymopoiesis and an increased production of naïve T cells (202). Furthermore, in young mice, the overexpression of Foxn1 resulted in a delay in thymic involution (204). Conversely, reducing Foxn1 expression levels in young mice induced premature thymic involution (205, 206). Further, it was shown that Fgf7 (also known as keratinocyte growth factor (KGF)) signaling increases the rate of thymic epithelial cell proliferation both at steady state and after thymic insult, such as infections or chemotherapy in rhesus macaques and mice (207). Furthermore, fetal thymic mesenchyme produces FGF7 and FGF10 that promote TEC differentiation and proliferation (208, 209). Additionally, TEC-derived FGF21 and the age-dependent downregulation thereof was implicated in mediating thymic involution (210). Age-related reduction of thymic tissue results in a decrease in the number of peripheral T cells and a reduction in T cell diversity. Differentiated αβCD8^+^-T cells, CD4^+^CD28^–^T cells and memory T cells subsequently accumulate (211, 212), which is a common characteristic of patients with chronic inflammatory diseases such as SLE, rheumatoid arthritis, multiple sclerosis or Graves’ disease (213, 214).

Abnormal thymus atrophy can be caused by infections, stress or medical interventions and lead to reduced thymopoiesis and a reduction in the generation of naïve mature T cells (215). Pharmacological application of synthetic glucocorticoids can also lead to thymic atrophy by engaging with intracellular glucocorticoid receptors (GRs), which trigger the activation of the mitochondrial apoptotic pathway in double-positive (DP) thymocytes (216, 217). Thymus hyperplasia can be caused by two forms of thymic enlargement, either due to thymic hyperplasia of normally organized thymic tissue caused by chemotherapy, corticosteroid use, irradiation or thermal burns or due to thymic lymphocytic hyperplasia which is characterized by increased number of lymphoid follicles with germinal centers in the thymus. Thymic lymphocytic hyperplasia has been associated with the development of autoimmune diseases such as myasthenia gravis and Graves’ disease (218). Another form of thymus enlargement can occur due to thymoma, which are often benign epithelioid neoplasm, which are also associated with the development of autoimmune conditions such as myasthenia gravis and pure red cell aplasia (219–222). Thymomas are classified based on morphological features and the content of lymphocytes into type A, type B (B1-B3) and micronodular thymoma with lymphoid stroma (MNT) which are distinguished from thymic carcinoma (TC) (223). Epithelial cells of thymomas show reduced expression levels of MHCII and Aire (224), thereby leading to inefficient T cell selection and the development of autoreactive lymphocytes (225). In a recent study, 116 thymomas from patients of which 34 developed myasthenia gravis were analyzed for myasthenia gravis specific characteristics using bulk- and single cell RNA-sequencing (226). A development of atypical immune microenvironments in the thymus with germinal center formation, B cell maturation, and ectopic neuromuscular expression of *GABRA5, MAP2, NEFL, NEFM, SOX15* by neuromuscular mTECs (KRT6^+^ GABRA5^+^ nmTECs) was described for MG-associated thymomas (226, 227). In this case the authors suggest that the presentation of neuronal autoantigens by mTECs in this scenario might lead to the development of autoimmunity with GC formations and affecting thymic B cell maturation in line with previous reports (227, 228), but in difference to the general dogma of self-antigen expression in mTECs for negative selection (i.e. deletion via apoptosis) of autoreactive T cells. Further analysis of the implications of thymic antigen expression in the formation of thymic GCs will be needed to analyze possible connections.

## 4 Thymic infections and autoimmune diseases

Among the environmental influences believed to potentially initiate the onset of autoimmune diseases, infections with pathogens in particular bacterial and viral infections are major suspects. The connection between viral infections and the initiation of autoimmune reactions through mechanisms such as molecular mimicry, bystander activation and epitope spreading have been proposed and recently reviewed elsewhere (229). Further, peripheral infections with pathogens such as gastrointestinal microbiota can cause thymus atrophy. Similarly, thymic atrophy is also known to occur after lipopolysaccharide injection which is used as a model system to study the mechanisms in mice (230). However, also thymic infections with viruses or bacteria are reported to cause thymic atrophy due to impaired thymocyte development and increased apoptosis of thymocytes (215). Even though the event of a direct infection of the thymic tissue is infrequent and a blood-thymus barrier similar to a blood-brain barrier has been proposed in the past and the selectivity and restrictiveness is controversially discussed (231–233), thymic infections with viruses and bacteria such as *Mycobacteria* have been described (234). Especially with regards to the recently identified thymic Tuft cells, which in mucosal tissues, are involved in pathogen clearance, a role of thymic tuft cells in the immune response towards thymic infections requires further investigations.

Thymic viral infections have been reported to disrupt T cell maturation and selection, suggesting them as potential causes for autoimmunity. Infection of the thymus with human and mouse roseoloviruses, belonging to the herpesvirus family, results in a temporary reduction of CD4^+^ SP and CD4^+^CD8^+^ DP thymocytes (235, 236). Furthermore, neonatal infections with roseoloviruses in mice have shown a connection to the development of autoimmune gastritis. Twelve weeks post-infection, these mice exhibited autoimmune characteristics, including stomach inflammation characterized by the production of autoantibodies and autoreactive CD4^+^ T cells (237). Notably, after such neonatal roseolovirus infections in mice, there is a transient decrease in numbers of medullary thymic epithelial cells (mTECs) and dendritic cells (DCs), along with diminished expression of Aire and tissue-restricted antigens (TRAs). The Coxsackievirus B (CVB), particularly the enterovirus strain CVB4 E2 is one of the viral environmental risk factors for the development of diabetes mellitus type 1 (DMT1) by influencing the inflammatory milieu, inducing bystander activation of CD8^+^ T cells and possibly through mechanisms of molecular mimicry (229). Thymic infections with CVB were investigated as a possible cause for the development of DMT1 using *in vitro* TEC systems and *in vivo* orally-inoculated mice (238–240).

## 5 Discussion

Therapeutic interventions targeting thymic tissue homeostasis and restoring thymopoiesis is often discussed as one of the aims to circumvent inflammaging and age-associated appearances of diseases (241). Perturbations of thymopoiesis due to infections, stress or interventions such as chemotherapy or irradiation are of high clinical relevance and much effort has gone into research to develop strategies which allow for a renormalization of thymic tissue homeostasis and thymopoiesis. Then again, an interference with the physiological age-related involution has to be carefully considered weighing the risk for the development of leukemic transformed T cells or autoimmunity (197). In this context, the relation of haematopoietic progenitor cell availability and the proportion of thymic epithelial compartments providing the niches for thymopoiesis needs to be considered. Different pre-clinical and clinical trial studies assessed thymic epithelial cell regeneration as one possible angle for thymus rejuvenation. One of the factors known to influence TEC maintenance is KGF. Recombinant human KGF was shown to enhance the regenerative capacity of the thymic epithelial compartment and showed cytoprotective effects (242). In a clinical trial study (NCT00071240), the regenerative effect of growth hormones was analyzed. The human recombinant growth hormone somatropin was administered in HIV-infected patients and was shown to increase thymic cellularity, naïve CD4^+^ and CD8^+^ T cells and peripheral immune responses (243). In a different clinical trial, known as the TRIIM (Thymus Regeneration, Immunorestoration and Insulin Mitigation) Study, healthy male participants were administered a combination of recombinant human growth hormone (rhGH), dehydroepiandrosterone (DHEA), and metformin. This regimen led to enhanced thymic function, beneficial immunological alterations, and improved risk profiles for several age-related diseases (244). Another ongoing trial (NCT04375657, TRIIM X trial) is currently underway and will analyze the potential therapeutic benefit of recombinant growth hormones in men and women. Other clinical trials analyzed the therapeutic potential of recombinant human IL-7 administration or sex steroid ablation to increase thymopoiesis (215). Further, ex vivo approaches and *in vitro* methods to enhance thymus function and generate transplantable thymic organoids are explored for their putative therapeutic potential (245, 246). During T cell development many thymocytes die in the course of positive and negative selection. At steady-state the presence of apoptotic thymocytes was shown to suppress the production of the regenerative factors IL-23 and BMP4 through TAM receptor signaling and activation of Rho-GTPase Rac1 and NOD2-mediated suppression (247). Upon thymocyte depletion the suppression is lacking and the production of IL-23 and BMP4 is increased. Interestingly pharmacological inhibition of Rac1 led to an enhancement in thymic function after acute damage offering possible treatment strategies for patients with compromised thymic function (247).

## Author contributions

FS: Visualization, Writing –review & editing. LH: Data curation, Visualization, Writing – review & editing. KR: Conceptualization, Supervision, Visualization, Writing – original draft, Writing – review & editing.
